# Using virtual environments to facilitate refugee integration in third countries

**DOI:** 10.1007/s10055-022-00659-x

**Published:** 2022-07-22

**Authors:** Mark Kirya, Kurt Debattista, Alan Chalmers

**Affiliations:** Warwick Manufacturing Group (WMG), University of Warwick, Coventry, CV4 7AL UK

**Keywords:** Virtual reality, Virtual experiences, Media, Knowledge transfer, Memory

## Abstract

Virtual experiences (VEs) have significant potential to enrich emotional interactions, to encourage socialisation and improve communication. In education, VEs offer new approaches for delivering content. In this paper we consider the application of VEs for assisting refugees in Senegal to learn how to navigate the complexities of the UK health system; a substantial stumbling block for their integration into society and for their own health. Participants ($N=122$), refugees awaiting to be repatriated, were exposed to material presented via three different media text, 360° videos and virtual reality (VR) across a total of seven different modalities. The experiment investigated specific attributes of the media that would facilitate refugees’ integration, such as knowledge received and retained, experience, usability and presence. The results show that interactive media, in particular VR, was significantly better across all tested attributes.

## Introduction

VR can naturally permit users to experience real-world environments, with their complexities, within virtual worlds where humans can perform real-world activities in a safe, controlled and repeatable manner.

Presenting information in an interactive manner can result in a more fulsome and efficient use of memory. To attain the best possible effect, it must be underpinned by a naturalness (realism) of the interaction; a believable resemblance that the user is interacting with the real world (Nowak and Biocca 2003).

Interactions have previously been shown to be effective in training, and knowledge and skills transfer (Carlin et al. 1997). VR can draw users into habitation of virtual situations that leads to deeper than usual engagement and enhances their ability to remember and therefore replicate the knowledge attained. It offers a unique way to expose individuals to controlled social environments (Veling et al. 2014) and involve them in a level of interaction conducive for learning and exchange of knowledge.

This paper addresses the difficulty that refugees encounter when navigating healthcare in their new countries. In particular, this work focuses on the case of UK-bound refugees and access to the UK’s National Health Service (NHS). Navigating the NHS is complex and requires a lot of knowledge in order for a person to be able to manage tasks, such as efficiently arranging a doctor’s appointment (Kang et al. 2019).

An experiment was conducted in which 122 refugee participants in Senegal who had yet to arrive in the UK, were exposed to three different media of knowledge transfer and asked to judge the experience, assess the level of engagement and usability, and undertake an assessment test to ascertain the extent to which they could successfully remember the knowledge attained. The three different media were a descriptive text, (the most commonly used mode of communication with refugees), a 360° image (a relatively new medium of communication) and an interactive virtual environment which was found to be completely new to the refugee participants.

**Fig. 1:**
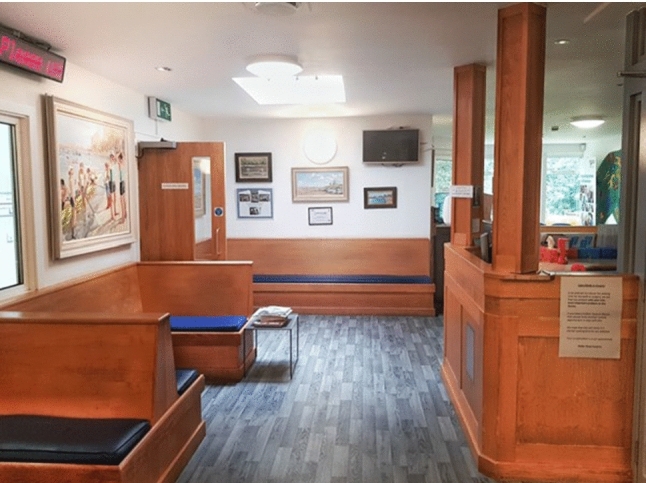
Photograph of the Glebe Road Surgery used in the design of the VR environment and 360 captures

The Glebe Road Surgery (Fig. 1) was used as a representative example of an NHS surgery. A text description of a surgery in London was written and presented on A4 paper. In addition, 360° images of the surgery were taken. A virtual reconstruction of the Glebe Road Surgery was also created using 3D laser scanning and digital photogrammetry.

Generalised information related to how to contact your GP, a waiting area, a play area for children, and how appointments are managed within the surgery, among other things were included in the text, 360° images and the VE. These features are the same in most health facilities across the UK.

Three variations of the simulation were used to assess the experience with the 360° and Virtual Environments. The impact of visual fidelity was studied by comparing results obtained by using an HTC Vive Pro head mounted display (HMD) compared to the lower quality HTC Vive HMD. Different sets of participants (seven) experienced the different media in order to compare results between different groups.

To the best of our knowledge this is the first study of its nature evaluating the performance of VR as a medium of knowledge transfer against other media, among refugees.

The main contribution of this work are:

- investigating the use of different media to make refugees more familiar with environments they will encounter
- an experiment with 122 refugees comparing across the different media
- significant results demonstrating the potential of interactive media, in particularly VR to, potentially, aid the process of integration with a more fulfilling experience.

The paper is divided into a further six sections. Section 2 discusses background for both refugees and VR and related work done in this area, while Section 3 discusses the materials. Section 4 presents the methodology, design and selection of participants. Section 5 presents the results of the experiment Sect. 6 discusses the findings, while Sect. 7 concludes and gives suggestions for future work.

## Background and related work

This section focuses on some of the key reasons behind refugees’ failure to integrate and why the use of appropriate technologies such as VR for knowledge transfer can be useful for such cases.

### Refugees integration

The UK Home Office surmises that integration “takes place when refugees are empowered to achieve their full potential as members of British society, to contribute to the community and to become fully able to exercise the rights and responsibilities that they share with other residents” (Herrick 2005).

The loss of social capital, knowledge, the language advantage, and other forms of resources during flight is a major destabilising factor for refugees as it sets them back and affects their ability to fully engage with society (Goodson and Phillimore 2008). It can also negatively impact their mental health and well-being especially because it is critical to the acquisition of other forms of capital which also aid integration. A further challenge is that integration in the UK , over the years, has been reduced to a one-way process wholly dependent on the refugees and their ability to fully embrace *Britishness* (assimilation). It thus places the burden of integration on refugees and immigrants alone which can hinder integration.

Chiswick (2016) focused on refugees who, on arrival, do not know their host country’s language and how that affects economic integration, and can result in the formation of immigrant enclaves divorced from the host country labour market. Language proficiency therefore becomes vital for their economic success as a form of human capital, and an economic good that is useful professionally, personally, and socially.

The most commonly used method of conveying messages to migrants and refugees, particularly about the UK National Health Service is through descriptive representations.

The work presented here posits that the use of VR, not only impacts the well-being of refugees as postulated by Smith (2016) but could also supplant the challenges imposed on them by language limitations and the feeling of powerlessness when faced with obstacles such as navigating the NHS.

### VR and memory retention

Morie et al. (2005) investigated human performance within a virtual environment. They showed that unconstrained “free will” exploratory behaviour is essential to research questions that involve the relationships between physiology, emotion, and memory. They also found that VR’s ability to simulate the complexity of real-world environments, makes it usable in neuropsychological assessments.

**Fig. 2:**
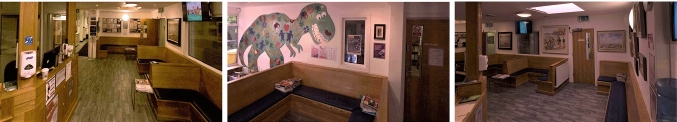
Views taken from the 360° image taken at the Glebe Road Surgery

Pimentel and Teixeira (1993), focused on the immersive VR interactive experience, in which the actual technology is completely forgotten, allowing VR to give the user a “sensory-rich interactive experience”, which impacts their uptake of information (Pimentel and Teixeira 1993). Sauzéon et al. (2012) also determined that being able to navigate in VR has an effect on memory and ensures better memory retrieval (Fig. 2).

A key goal of the research presented in this paper is to explore the efficacy of VR technology in the transfer to, and retention of knowledge by refugees based on the hypothesis that VR is more efficacious because of its ability to elicit the engagement of its user.

Knowledge transfer is the integration of skills acquired during an experience into daily practice at a time after the experience (Cowan et al. 2015). Contextualising this transfer to intersect with actual practice achieves better outcomes and gives importance to the appropriateness of a medium of transfer (Cowan et al. 2015).

The introduction of user-friendly technology in knowledge transfer can enhance current traditional media (such as descriptive texts) with their numerous limitations. Research shows that interactive and collaborative forms of knowledge transfer are more effective in inducing changes in application (Achuthan et al. 2017).

VR is an enhanced form of depictive representation which goes one step farther than the previous two by offering interactivity, generating a more wholesome and easily repeatable experience (Schnotz 2005).

VR can portray settings, characters and actions in a way that captures the attention of the user, while at the same time simplifying contextual complexities and challenges (Choi and Johnson 2007).

Users with no prior knowledge or experience do not have to formulate abstract perceptions as they are able to benefit from the technological systems such as VR to enhance their understanding.

### VR effectiveness

Ruggeroni (2001) studied the use of VEs in knowledge transfer with a specific focus on the influence of immersive subjective tendencies on the transfer of knowledge in the educational process. He found that although the knowledge transfer was not strongly affected by immersive tendencies, a low level of interaction could significantly affect the transfer of knowledge.

Palanica et al. (2019) specifically focused on the limited understanding that most patients exhibit with medical procedures. According to them, patients, who are typically short on scientific knowledge or background, have a hard time understanding what their healthcare providers tell them about medical procedures or surgeries. They therefore studied the use of VR in helping patients to understand treatment information by use of VR technology designed to help facilitate this knowledge transfer between physicians and patients. They found that because the experience was engaging, it helped patients to learn about complicated medical information in part because it allowed them to interact with representations of their own anatomy and procedural findings, rather than being passively told about their operation; it helped patients visualize and better understand their disease.

Alfalah et al. (2019) studied the use of VR as a medical training tool to improve the quality of medical skills of medical students, specifically on heart anatomy. They focused on a comparative study between traditional medical teaching modalities and VR technology. The results showed that VR enhanced experiences and improved their understanding of heart anatomy. There was a higher satisfaction rate for VR in relations to structure and visualisation.

Abich et al. (2021) reviewed literature to ascertain the effectiveness of VR-based training. They found that VR-based training improves psychomotor performance, knowledge acquisition, and spatial ability.

Kaplan et al. (2021) could not identify the superiority of extended reality (a combination virtual Reality, augmented reality and mixed reality) over training in a non-simulated control environment. They also noted that current literature on the effects of Virtual Reality, augmented reality and mixed reality for training enhancement is sparse. They point to the importance of choice of variables in arriving at useful conclusions, and the importance of highlighting individual differences among population cohorts as well as the need for performance comparisons between population cohorts. According to them, this is critical to understanding differences in results, attributable directly to the effect of population factors such as experience or comfort with technology. They also talk about the range of headsets, of different quality, which could impact studies.

**Fig. 3:**
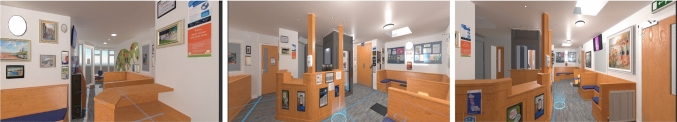
Virtual images of the Glebe Road Surgery created through photogrammetry and laser scanning

Woon et al. (2021) researched the effectiveness of VR training in improving knowledge among nursing students. They found that VR training was more efficacious in delivering procedural knowledge to undergraduate nursing students when conducted in multiple, self-guided, short sessions within 30 min and by using low–moderate level of immersion (Fig. 3).

## Motivation

Migration and population mobility in Europe have increased the need for information dissemination and transfer of knowledge to new migrants to help them navigate socio-economic systems (Braun et al. 2013) and thereby more easily integrate into their new host environment. Elements of Europe’s socio-economic systems are novel to most migrants. As such, they have limited knowledge on how to navigate them. One of the harder challenges is access to the healthcare systems of such countries (Kang et al. 2019).

Traditionally, a common way of enhancing their knowledge is through descriptive text and image depictions, for example via specific websites. However, it is not yet clear how best to provide the refugees with the information they both need, and that is fully actionable.

This work investigates alternative means, around digital media, particularly VR, that can be used to transfer knowledge to refugees and attempts to ascertain which one is better to help ensure knowledge understanding and retention before they travel to the UK.

## Method

This section presents the methodological aspects of the experiment. The experiment involves comparing three methods of presenting the information: text, 360° images and VR. The main objective is to investigate the efficacy and impact of each of the approaches on knowledge transfer. More specifically it aims to evaluate the hypothesis that VEs are highly effective in facilitating ”remembering” and are therefore better suited to helping refugees integrate in third countries.

### Design

The overarching objective of the experiment was to ascertain how media impacts memory and behaviour. To achieve this, a descriptive text of a surgery (Glebe Road Surgery in London, UK) was presented on an A4 piece of paper. In addition, 360° images and a virtual representation of the same surgery were captured and created, respectively. Participants were randomly divided into seven groups based on the different media (text, 360° images, and Virtual Environment) and factored based on fidelity using a desktop, an HTC Vive HMD, and the HTC Vive Pro HMD.

The choice of media is a between-participants independent variable (IV) for the experiment. It was disaggregated into seven different factors as follows, where the term in brackets denotes how the factors will be referred to in the rest of the article:

- **Descriptive Text (Text)**
- **360**°** Images on:** Desktop (360D), Vive (360V) and Vive Pro (360VP)
- **VE on:** Desktop (VED), Vive (VEV) and Vive Pro (VEVP)

The dependent variables (DVs) were:

- **Experience**: Denoting participants’ experience using the seven different media.
- **Presence (Measure of Presence):** The extent to which participants felt engaged by the medium.
- **Test1:** Assessment test for knowledge transferred administered immediately after the experiment.
- **Test2**: Knowledge retention assessment test administered between 10 - 14 days after Test1, using the same questions (translated into French because most of the participants were francophone).
- **Usability:** Participants assessment of ease with which they could use the specific medium.

Initially, three different sets of questionnaires (on Experience, Presence and Usability) were administered for each of the DVs and were based on a multi-item scale where one was the lowest score and seven, the highest score. Participants were asked to fill out which scale best comported with their own experience. There were 32 questions on Presence and six questions each on Experience and Usability.

The questions on experience were derived from the Presence and the Immersive Tendencies Questionnaires (Witmer and Singer 1998), while the usability questions were derived from the Post-Scenario System Usability Questionnaire (Lewis 1992) and Lund’s Usefulness, Satisfaction, and Ease of use Questionnaire (Lund 2001).

Participants were exposed to the experiment and then asked to complete the assessment questions from which their responses were evaluated (Test1 and Test2). The assessment questions, reviewed by experts in the psychology and medical field, included a 14-question test on the subject matter (access to the Glebe Road Surgery), five of which were aimed at testing their application of the knowledge (navigation of the surgery). These were also marked out of seven (with one lowest scores and seven the highest). Participants were scored based on their grasp of the concepts presented. The participants were then asked to return 10–14 days after completing the experiment and take the 14-question assessment test a second time (Test2) to assess their memory retention.

Overall, the underlying hypothesis of this study (H1) is that VR, compared to other media (descriptive text and 360° images), is the most efficacious in understanding and retaining presented information.

### Participants

One hundred twenty-two refugees volunteered to take part in the experiment. A non-probability sampling method was used to determine the sample size for the study. The sampling frame for this study were refugees with university level education with basic IT knowledge. The participants had no previous experience with VR and knew nothing about the UK National Healthcare System or the Glebe Road Surgery. All 122 participants were exposed to just one of the seven different modes of knowledge transfer resulting in 122 different results per DV. All seven variations of the media were conducted by different participants as follows:

1. Text: $$N = 28$$
2. 360D ($$N = 14$$), 360V ($$N = 14$$), 360VP ($$N = 19$$)
3. VED ($N = 14$), VEV ($N = 14$), VEP ($N = 19$)

The selection of participants per variation was randomised. The higher numbers for 360 and VE represent the three different media variations. Given the sample size, the investigators surmised that the slight differences in sample space per media variation did not have a significant effect on the results.

#### Characteristics of participants

Forty-eight females and 73 males were recruited for the experiment. Over 95$\%$ of the participants were between the ages of 21 and 30 years. The participants were refugees from six different countries (Central African Republic, the Democratic Republic of the Congo, the Republic of Congo, Gabon, Cameroon, and Cote d’Ivoire). They were recruited on voluntary terms by word of mouth, use of leaflets and by speaking to their leaders and the UN Refugee agency.

### Materials

This section discusses in detail how photogrammetry and laser scanning were used in developing the virtual environment and how the 360° images were captured. All images (real or virtual) are related to the Glebe Road surgery at *1 Glebe Rd, London SW13 0DR, United Kingdom*.

#### Photogrammetry and laser scanning

Photogrammetry and high-quality 3D laser scanning were used to obtain relevant information about the interior of the Glebe Road Surgery.

The laser scan point cloud and photographs were used to create the environment with millimeter accuracy. The images were used to create physical-based render textures, to accurately recreate the environment and lighting conditions. The models were done in Autodesk Maya, and the Unity 2018.4 engine was used to create a VR build of the surgery using the SteamVR plugin. The build was tested and ran flawlessly on the HTC Vive and HTC Vive Pro headsets.

#### Capturing 360° images

360° are 2D images taken by a high definition 360° camera, a Spheron SceneCam (full spherical HDR images of 360° x 180$^\circ$, over 100 megapixels). They were processed using SpheronLite software, uploaded on a desktop and displayed on a HTC Vive and the HTC Vive Pro Head Mounted Displays. The quality levels of both the VR and the 360° images were high. The images were displayed on a computer monitor in order to make a distinction with the display on the HMDs in terms of quality, and the ability to heighten participants’ level of engagement. The HTC Vive Pro displayed a superior quality of images with a screen resolution of $1440 \times 1600$ pixels per eye (or combined resolution of $2880 \times 1600$ pixels) compared to the HTC Vive screen resolution of $1080 \times 1200$ pixels per eye (and combined resolution of $2160 \times 1200$ pixels).

For the 360° conditions the participants could only spatially navigate in a restricted space (restricted to the points where it had been captured). The navigation in the VR on the other hand was unrestricted which was generally reflective of the characteristics of the specific medium used. The participants did not complete any specific task but simply conducted a spatial navigation of the virtual environment and the 360° images.

Administration of the experiment was done by text, 360° images and VR. The text component of the experiment was administered using A4 pieces of paper on which a description of the Glebe Road Surgery was provided. Participants were asked to read the text and respond to related questions thereafter (prepared in French since most of the participants were francophone).

The 360° and VR components, were administered three different media differentiated based on the fidelity including a desktop computer connected to a 4K high Definition 55” screen for maximum high-definition effect, an HTC Vive HMD with handheld controllers, and an HTC Vive Pro to inject sound in addition to the visual and hand-controlled effects.

### Procedure

As a control measure and to minimise practice effect, participants were contacted at least three days prior to the experiment and asked to arrive within a specific time slot. On arrival, they were taken through the COVID-19 protocols which included testing their temperature, sanitising their hands and handing them a pair of surgical gloves to use while manipulating equipment and to ensure safety in the event of contact with any materials or fixtures inside the room where the experiments were conducted. At this point it was randomly decided which participants would use which media. The investigators had three assistants who helped to assign participants to the different media variations at random.

The experiment was conducted in three separate rooms of $20 \times 20$ m for those using the HMDs, and $5 \times 5$ m each for those using the desktop computer and reading the descriptive text.

Participants were then given instructions (prepared in French as most of the participants were Francophone) on how the experiments would be conducted. The written instructions were read verbatim (in French) to all participants to ensure that they all received the same information.

The participants who took the text component of the experiment were immediately allowed to read through the descriptive text and thereafter respond to the assessment questions (prepared in French). The remaining participants had to go through a 5-min trial of the 360° images and the virtual environment on a desktop and using the HMDs. They were then exposed to a 15 to 20-min experience of the 360° images and virtual environment of the Glebe Road surgery.

The 360° images and virtual environment were displayed on a desktop computer, and the HTC Vive and HTC Vive Pro HMDs as previously mentioned. They were then given a 10-min reorientation break before responding to the 14-question assessment to test their knowledge attainment. After a 10–14-day period, the second knowledge retention test was administered without recourse to the material provided previously to assess the participants ability to remember what they had been shown.

Participants were encouraged to respond objectively to all questions, which were designed to give them the opportunity to answer the questions in their own words. The questions were designed to find evidence that on the one hand a transfer of knowledge had occurred, and on the other, there had been knowledge retention. The Presence and the Immersive Tendencies Questionnaires (Witmer and Singer 1998) tested their level of engagement and ascertained what was responsible for that level of engagement and the quality of their experience.

The two questionnaires were based on a 7-point Likert scale to measure the immersive and engagement potential of each individual and the degree to which individuals were able to ignore outside distractions. The intention here was to analyse engagement/immersion as a subjective tendency in relation to the transfer and retention of knowledge.

#### The Experiment

The entire experiment took between 90 and 120 min to complete. There was then a 20-min break to sanitise all equipment and fixtures before the next group of participants were allowed in. The arrival of participants was carefully choreographed by inserting a 15-min interval between groups, to minimise contact between the outgoing and incoming participants.

At the end of each session, participants were requested not to discuss any element of the experiment amongst themselves and to return in 14 days to take the “knowledge retention test”. They were also debriefed to ascertain if they experienced any harm or discomfort during the experiment, to discuss the expected outcomes of the research and how they would be deployed, and to answer any questions they might have.

## Results

In order to analyse the effect of the media on knowledge transfer and retention, the results of the seven factors were evaluated based on the seven different groups of participants, each of which were exposed to only one factor of the experiment.

For all DVs we present results for descriptive and inductive statistics. Depending on an initial analysis of the distribution we conduct either parametric ANOVA or the nonparametric equivalent.

### Experience

**Fig. 4:**
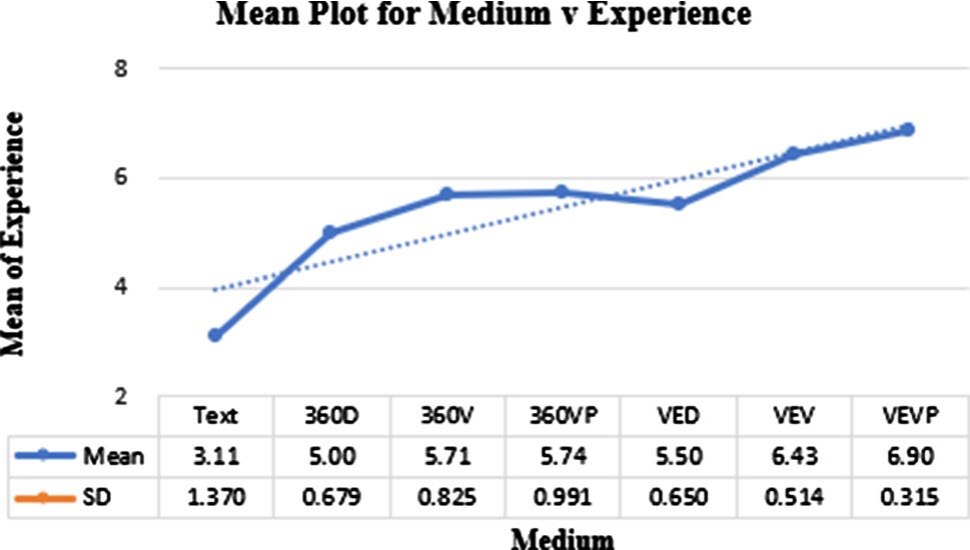
Means plot for the media different factors against experience

Figure 4 plots the means across the factors. On average, across all groups, $\mu = 5.14$ with $\sigma = 1.501$. Individually, the seven factors scored as highlighted in Fig. 4.

Kolmogorov–Smirnov test for this DV was significant $p< 0.05$ indicating a non-normal distribution. The nonparametric Kruskal–Wallis test showed a main effect of Experience was significant $H(6) = 82.91$, $p < 0.001$.

Pairwise comparisons with Bonferroni corrections are shown in Table 1. The table presents pairwise comparisons for all DVs. The pairwise comparisons were either parametric or nonparametric depending on the DV. These are indicated in the text of each DV. The factors are sorted by their means (where the leftmost factor has the best mean for that DV). The colour groupings demonstrate lack of significance in the pairwise comparisons.

The means plots and pairwise comparisons showed a significantly superior mean for VEVP, followed closely by VEV. The other media apart from Text do not exhibit any significant differences. The display on HMDs seem to dominate, with 360VP having a higher mean than VED too, albeit with no significant differences shown.

### Presence

**Fig. 5:**
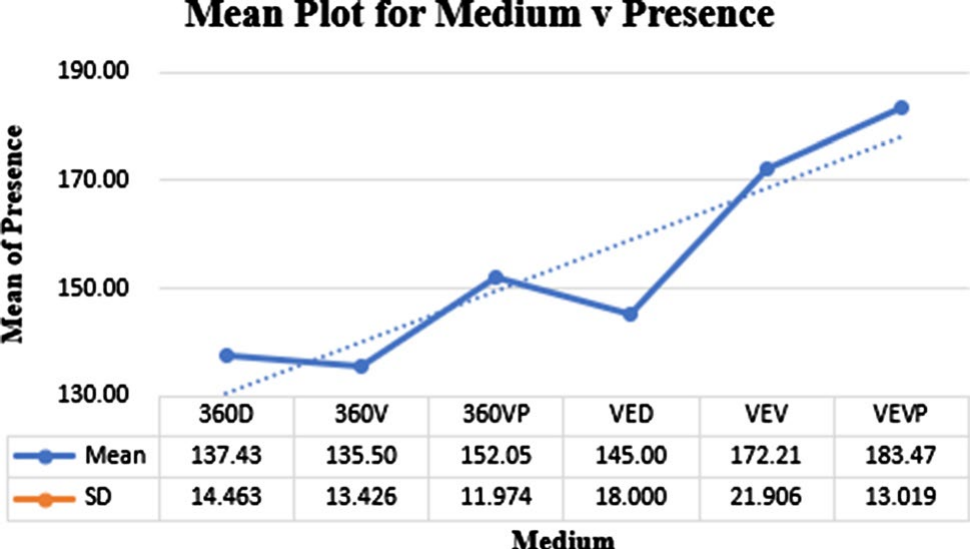
Means plot for the different media factors against “measurement of presence”

A plot across means is shown in Fig. 5. The group mean score was $\mu = 122.47$ and $\sigma = 53.14$ and the individual scores were as highlighted in Fig. 5 (note that the text was not measured for Presence).

Kolmogorov–Smirnov showed non-normal data across the media ($p< 0.05$). Kruskal–Wallis showed the main effect or Presence was significant, $H(6) = 98.19$, $p < 0.001$.

Pairwise comparisons can be seen in Table 1. Results show VEVP and VEV elicited a higher level of presence than the rest of the media.

### Tests

Both Test1 and Test2 demonstrate a normal distribution under all media ($p > 0.05$ for Kolmogorov–Smirnov test). Results for the knowledge tests are initially conducted via a factorial 2 (tests) $\times$ 7 (media) mixed-measure ANOVA. Media is the between-participants variable consisting of the seven media and tests is the within-participants variable consisting of the two test DVs (Test1 and Test2). The main factor of test is significant $F(1, 115) = 304.216$, $p < 0.01$. The main factor of media is also significant $F(6, 115) = 13.546$, $p < 0.01$. The interaction tests $\times$ media is also significant $F(6, 115) = 11.963$, $p < 0.01$. Due to the significance in the main factors and the interaction we conduct more detailed analysis in the following. We thus rejected H0 and accepted *H*1.

#### Test1

The univariate one-way ANOVA of Test1 assessed the effect of the medium on the attainment of information/knowledge from the experiment.

**Fig. 6:**
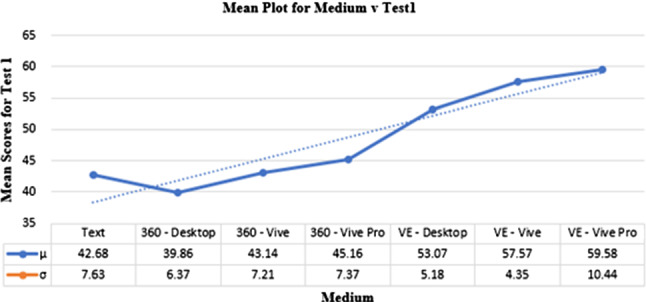
Means plot highlighting performance of participants in Test1 with the different media factors

The main effect of Test1 was statistically significant, $F (6,115) = 31.78$, $p < 0.001$. Figure 6 shows the mean scores across Test1. The group mean score was $\mu = 48.33$ and $\sigma = 10.240$ and individual scores were as highlighted in Fig. 6.

Pairwise comparisons with Bonferroni corrections can be seen in the Test1 row of Table 1. These results demonstrate the superiority of VE on the knowledge attainment test. However, VED and VEV have lower standard deviations ($\sigma = 5.181$ and $\sigma = 4.345$) than VEVP ($\sigma = 10.442$) and the other factors (Text: $\sigma = 7.626$, 360D: $\sigma = 6.371$, 360V: $\sigma = 7.21$, 360VP: $\sigma = 7.366$) demonstrating a relative stability of the results and therefore a superiority of Virtual Reality.

**Table 1:**
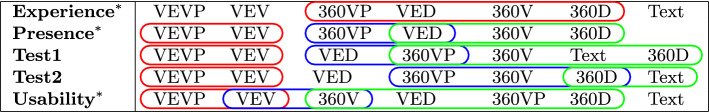
Pairwise comparisons, with Bonferroni corrections, between the different factors for all DVs

All factors are sorted left to right by mean. Coloured groupings denote no significant difference amongst factors in a group. $^{*}$ denotes use of non-parametric statistics. A clear, significant, preference for the virtual reality media emerges

#### Test2

**Fig. 7:**
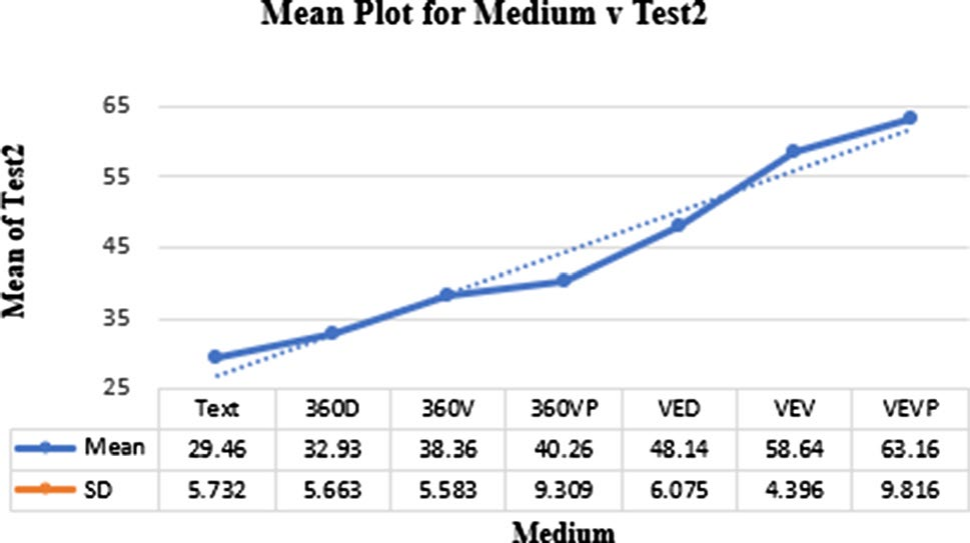
Means plot highlighting correlation between media factors participants’ performance in Test2

A one-way ANOVA was conducted for Test2. The main effect was statistically significant, $F (6,115) = 61.84$, $p < 0.001$. Figure 7 shows a plot of the descriptive statistics. The group mean score was $\mu = 41.8$ and $\sigma = 12.191$ and individual scores are highlighted in Fig. 7.

Pairwise comparisons are shown in Table 1. The mean scores on the knowledge retention/memory test demonstrated the superiority of VE. As with Test1, the lowest SD related to VEV ($\sigma = 4.012$) demonstrating a relative stability of the results and therefore a superiority of VR.

#### Test1 and Test2

**Fig. 8:**
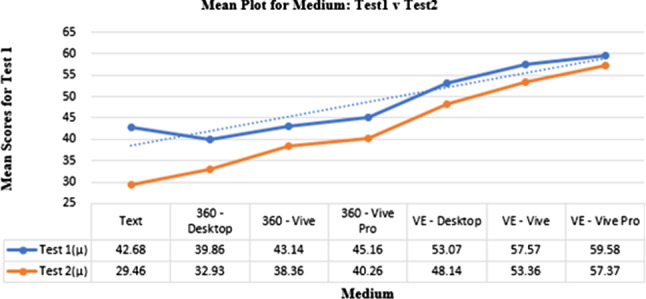
A comparison of means and trends between Test1 and Test2

Figure 8 shows a comparison between Test1 and Test2. Results demonstrated that participants had a 69$\%$ chance of retaining the knowledge they had received through the descriptive text method, a greater than 80$\%$ chance of recovering the knowledge transferred by the 360° image, and a greater than 90$\%$ chance of recovering the knowledge transferred by VR.

#### Usability

**Fig. 9:**
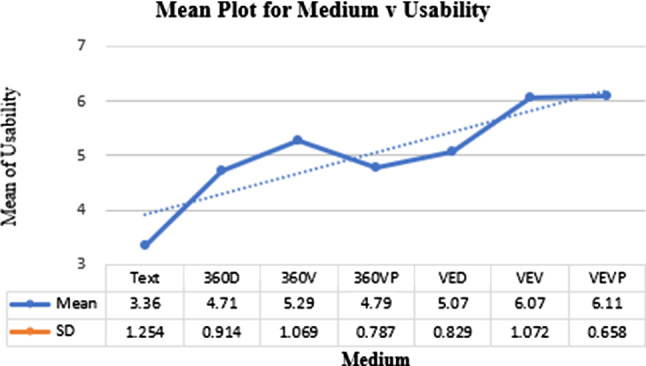
Means plot for the media different factors against usability

On average, across all groups, $\mu = 4.66$ with $\sigma = 1.265$. Individually, the scores for the seven factors are shown in Fig. 9.

Kolmogorov–Smirnov showed non-normal data for Usability ($p < 0.05$). Kruskal–Wallis results demonstrated the main effect of Usability was significant, $H(6) = 61.95$, $p < 0.001$. Pairwise comparisons showed best results for VEVP and VEV again.

## Discussion

This study showed important tendencies that knowledge transfer, experience, usability and presence were strongly impacted by the type of medium used and the level of realism presented.

On average, VR outperformed all the other variations most likely due to its inherent ability for interaction and engagement. The 360° came second and the text variation, third. The significant difference in performance of VEV and VEVP can be surmised as the import of fidelity or level of realism and its effect on the level of engagement. The effect of fidelity and interaction can also be seen in how 360VP consistently outperformed 360V and 360 and VED on some occasions. Overall, it could be argued that a lower level of engagement and the ”interactive-ness” of the text and 360° images may have had the biggest impact on their relatively lower scores on memory processing.

For refugees to better comprehend the descriptive text, and to appropriately apply that knowledge, they would have required prior knowledge of the NHS or the Glebe Road Surgery. This would have helped them to formulate mental models of what they were being asked to internalise. The same applies to the 360° images, where more images would have been required to provide a more wholesome story, and potentially fill ”information gaps”. The general lack of prior knowledge meant that participants who were exposed to the text and 360° images, possessed a poorer internal source of information which then made mental model construction from the information provided, relatively more difficult. On the other hand, the VE offered alternative routes for mental model construction. In particular, it did this through its inherent ability to bring the participants into the health system and thus create a lasting memory of what that system actually looks like, and the experience of navigating it.

The virtual environment was built at a cost of about GBP 5500. These costs represented the actual cost of a digital artist creating the virtual environment, as well as the cost of travel between the University of Warwick and the Glebe Road Surgery in London for data collection using the FARO Scanner. With regard to the 360° images, cost implications mainly related to the travel between the University of Warwick and the Glebe Road Surgery in London, to capture the images. The descriptive text was also developed after travel to London and so had nearly identical cost to the cost of capturing and rendering the 360° images. Three separate day-long travels (06h00–16h00) were made to the surgery in London to capture the images. Each, the trip had to be made on a Saturday when there were no people at the surgery. Although the VE was more expensive, once it had been captured, there was no need for a return trip. It was simply rendered based on images captured and accordingly improved. The 360° images on the other hand required three separate trips.

This work demonstrates the benefits of VR, and costs and the time required should come down as more straightforward capture methods, based on for example, photogrammetry, become more streamlined and software to support and generate virtual environments becomes more common place. We have added aspects of the above to the discussion. This view is also postulated by Abich et al. (2021).

## Conclusions and future work

According to our study, the use of virtual experiences offers the potential as an important knowledge acquisition tool for refugee integration. The obtained results indicated clearly that the VR could be used as a powerful knowledge transfer tool because of its superior ability to elicit formation of a memory.

Further studies will focus on other factors such as measurement of the level of realism, which in the current experiment was a non-factor. In addition, further research is needed into the correlation between fidelity, higher levels of engagement (immersion) and how the two could impact memory and perhaps changes in behaviour or a replication of knowledge attained. This could help highlight how a greater degree of engagement may assist to find new thresholds or intensities scales in relation to memory encoding.

## References

[CR1] Abich J, Parker J, Murphy JS, Eudy M (2021). A review of the evidence for training effectiveness with virtual reality technology. Virtual Real.

[CR2] Achuthan K, Francis SP, Diwakar S (2017). Augmented reflective learning and knowledge retention perceived among students in classrooms involving virtual laboratories. Educ Inf Technol.

[CR3] Alfalah SFM, Falah JFM, Alfalah T, Elfalah M, Muhaidat N, Falah O (2019). A comparative study between a virtual reality heart anatomy system and traditional medical teaching modalities. Virtual Real.

[CR4] Braun S, Slater C, Gittins R, Ritsos PD, Roberts JC (2013) Interpreting in virtual reality: designing and developing a 3D virtual world to prepare interpreters and their clients for professional practice. In: New prospects and perspectives for educating language mediators, pp 93–120

[CR5] Carlin AS, Hoffman HG, Weghorst S (1997). Virtual reality and tactile augmentation in the treatment of spider phobia: a case report. Behav Res Ther.

[CR6] Chiswick BR (2016) Tongue tide: the economics of language offers important lessons for how Europe can best integrate migrants. Finance Dev 53(003)

[CR7] Choi HJ, Johnson SD (2007). The effect of problem-based video instruction on learner satisfaction, comprehension and retention in college courses. Br J Educ Technol.

[CR8] Cowan Brent, Rojas David, Kapralos Bill, Moussa Fuad, Dubrowski Adam (2015). Effects of sound on visual realism perception and task performance. Vis Comput.

[CR9] Goodson LJ, Phillimore J (2008). Social capital and integration: the importance of social relationships and social space to refugee women. Int J Divers Organ Commun Nations.

[CR10] Herrick Christine (2005). Integration matters—a national strategy for refugee integration. Race Equal Teach.

[CR11] Kang Cara, Tomkow Louise, Farrington Rebecca (2019). Access to primary health care for asylum seekers and refugees: a qualitative study of service user experiences in the UK. Br J Gen Pract.

[CR12] Kaplan AD, Cruit J, Endsley M, Beers SM, Sawyer BD, Hancock PA (2021). The effects of virtual reality, augmented reality, and mixed reality as training enhancement methods: a meta-analysis. Hum Factors.

[CR13] Lewis JR (1992) Psychometric evaluation of the post-study system usability questionnaire: the PSSUQ. In: Proceedings of the human factors society annual meeting, vol 36. Sage Publications Sage CA, Los Angeles, CA, pp 1259–1260

[CR14] Lund AM (2001) Use questionnaire: usefulness, satisfaction, and ease of use. Retrieved 20 Sept 2019

[CR15] Morie JF, Iyer K, Luigi DP, Williams J, Dozois A (2005). Development of a data management tool for investigating multivariate space and free will experiences in virtual reality. Appl Psychophysiol Biofeedback.

[CR16] Nowak KL, Biocca F (2003). The effect of the agency and anthropomorphism on users’ sense of telepresence, copresence, and social presence in virtual environments. Presence Teleoper Virtual Environ.

[CR17] Palanica A, Docktor MJ, Lee A, Fossat Y (2019). Using mobile virtual reality to enhance medical comprehension and satisfaction in patients and their families. Perspect Med Educ.

[CR18] Pimentel K, Teixeira K (1993) Virtual reality through the new looking glass

[CR19] Ruggeroni C (2001) Ethical education with virtual reality: immersiveness and the knowledge transfer process. In: The communications through virtual technology: identity community and technology in the internet age, pp 110–133 (2001)

[CR20] Sauzéon H, Pala PA, Larrue F, Wallet G, Déjos M, Zheng X, Guitton P, N’Kaoua B (2012). The use of virtual reality for episodic memory assessment: effects of active navigation. Exp Psychol.

[CR21] Schnotz Wolfgang (2005). An integrated model of text and picture comprehension. Camb handb Multimed Learn.

[CR22] Smith A (2016) Creative English: balancing creative and functional language needs for adult refugees, asylum seekers and migrants. Scenar J Perform Teach Learn Res X(1):1–17. 10.33178/scenario.10.1.1

[CR23] Veling Wim, Brinkman Willem-Paul, Dorrestijn Emily, Van Der Gaag Mark (2014). Virtual reality experiments linking social environment and psychosis: a pilot study. Cyberpsychol Behav Soc Netw.

[CR24] Witmer BG, Singer M (1998). Measuring presence in virtual environments: a presence questionnaire. Presence.

[CR25] Woon APN, Mok WQ, Chieng YJS, Zhang HM, Ramos P, Mustadi HB, Lau Y (2021). Effectiveness of virtual reality training in improving knowledge among nursing students: a systematic review, meta-analysis and meta-regression. Nurse Educ Today.

